# Differential impact of liana colonization on the leaf functional traits of co-occurring deciduous and evergreen trees in a tropical dry scrub forest

**DOI:** 10.1007/s10265-023-01474-4

**Published:** 2023-05-31

**Authors:** Vivek Pandi, Kanda Naveen Babu, Ashaq Ahmad Dar

**Affiliations:** 1 Manipal Centre for Natural Sciences, Centre of Excellence, Manipal Academy of Higher Education (MAHE), Madhava Nagar, Manipal, Udupi, Karnataka 576 104 India; 2 Department of Ecology and Environmental Sciences, School of Life Sciences, Pondicherry University, Puducherry, 605 014 India; 3 Department of Ecology, French Institute of Pondicherry, Pondicherry, 605 001 India

**Keywords:** Acquisitive strategy, Chlorophyll a/b ratio, Leaf economic spectrum, Leaf nitrogen concentration, Shade-tolerance, Specific leaf area

## Abstract

The present study was carried out to analyze the leaf functional traits of co-occurring evergreen and deciduous tree species in a tropical dry scrub forest. This study also intended to check whether the species with contrasting leaf habits differ in their leaf trait plasticity, responding to the canopy infestation by lianas. A total of 11 leaf functional traits were studied for eight tree species with contrasting leaf habits (evergreen and deciduous) and liana-colonization status (with or without liana). In the liana-free environment (L^–^), evergreen trees had significantly higher leaf tissue density (LTD) and total chlorophyll (CHL_t_) than the deciduous species. Whereas the deciduous trees had higher specific leaf area (SLA) and mass-based leaf nitrogen concentration (N_mass_). The leaf trait-pair relationship in the present study agreed with the well-established global trait-pair relationships (leaf thickness (LT) vs. SLA, N_mass_ vs. LT, SLA vs. N_mass_, and LDMC vs. SLA). There was a significant difference between L^+^ and L^–^ individuals in leaf area (LA), petiole length (PL), SLA, LDMC, and CHL_t_ in the deciduous species. On the other hand, evergreen species showed marked differences across LT, SLA, LTD, N_mass,_ and chlorophyll components between L^+^ and L^–^ individuals of the same species. The results revealed the differential impact of liana colonization on the host trees with contrasting leaf habits. The deciduous species with the acquisitive strategy can have a competitive advantage over evergreen species in the exposed environments (L^–^), whereas evergreen species with shade-tolerant properties were better acclimated to the shaded environments (L^+^). Therefore, liana colonization can significantly impact the C-fixation strategies of the host trees by altering their light environment and further, the magnitude of such impact may vary among species of different leaf habits. The result also indicated the patterns of convergence and divergence in some of the leaf functional traits between evergreen and deciduous species explaining the patterns of species co-existence.

**Supplementary Information:**

The online version contains supplementary material available at 10.1007/s10265-023-01474-4.

## Introduction

Plants’ exposure to varying light environments is temporally dynamic. However, leaves invariably adapt to changing light conditions via structural and functional plastic responses (Bond et al. 1999; Yoshimura et al. 2010). Therefore, changes in the light levels to which a species has become acclimated can result in a variety of physiological responses in its biochemical, anatomical, and growth-related traits (Atroch et al. 2001; Taiz and Zeiger 2006). Several studies have looked into the morphological and physiological adaptations of vascular plant leaves to different light conditions during their development (Anderson et al. 1995; Kurasová et al. 2002). At the individual level, leaves acclimated to heterogeneous light environments are found within the canopy due to self-shading and shading from neighboring trees (James and Bell 2000; Wyka et al. 2012). The leaves that grow under direct sunlight are typically acclimated for high light environments (sunlit leaves) with a suite of traits favoring their performance under higher irradiance, while leaves growing in the shaded inner canopy adapt to low-light environments (shade leaves) (Lambers and Poorter 1992). The physiological differences between sunlit and shade leaves are usually due to the differences in leaf structure (Gratani et al. 2006). Leaves growing under shade are relatively thinner than sunlit leaves due to the under-development of mesophyll tissues (Grecco et al. 2014; Nobel 1976). In addition, sunlit leaves are more productive by means of higher light-saturated photosynthetic capacity (Olsen et al. 2002) than the shade leaves, which are characterized by innate lower photosynthetic rates, higher total chlorophyll, and lesser chlorophyll a/b ratio (Taiz and Zeiger 2006). Therefore, a decrease in the proportion of sun-shade leaves may decrease the carbon gain per unit leaf area. However, shade-acclimated leaves can be more efficient under low light environments, thanks to their specialized anatomical and physiological adaptations (Givnish 1988; Taiz and Zeiger 2006). Though sun and shade leaves have many physiological and morphological differences, it remains unclear how co-occurring plants with different leaf functional types differ in the magnitude of plastic responses to heterogeneity in the light environment.

The co-occurring evergreen and deciduous species with distinct leaf morphological and functional features (Huang et al. 2015) are ideal candidates to understand such variations in structurally and functionally essential leaf traits and their pattern of co-existence in the same environment. The evergreen leaf habits are associated with lower photosynthetic efficiency compensated by their extended leaf longevity, whereas deciduous species are short-lived with greater photosynthetic efficiency (de Souza et al. 2020; Kröber et al. 2015). Both functional types exhibit a trade-off between faster resource acquisition and increased foliar longevity (de Souza et al. 2020; Jiang et al. 2016; Wright et al. 2004). Earlier studies (Givnish 2002; Valladares et al. 2000) have shown that deciduous species with short-lived leaves can have better trait plasticity as a response to light variation than the evergreen species with higher leaf longevity, though such a trend may not be universal (Markesteijn 2007). Although much emphasis has been given to plant responses for self-shading and shading from neighboring trees, the light acclimation responses of host trees to the liana colonization have gained little attention.

Lianas are woody climbers that lack the mechanical strength needed for self-sustaining vertical growth. Therefore, they climb trees to reach the forest canopy and expose themselves to the well-lit environment. Since lianas do not need to invest heavily in mechanical tissues, they allocate a significant portion of their resources to foliage production. Several studies have found that lianas have a higher stem-leaf ratio than trees of comparable girth/diameter. Lianas often form a dense carpet of leaves over the host trees’ crown (Avalos et al. 2007; Ichihashi and Tateno 2015; Van der Heijden et al. 2013), limiting or leaving no light for the trees to perform photosynthesis Furthermore, liana colonization on host trees is becoming more common in the tropics. Gerwing (2001), for example, reported that 90% of the trees (15.7 cm gbh) in a seasonal evergreen forest in Brazil carried at least one climber. This increased liana colonization rate, particularly in the tropics, will have profound consequences for overall forest carbon assimilation rates.

The belowground competition of lianas and trees for water and nutrient uptake has been well studied (e.g., Pérez-Salicrup and Barker 2000; Pérez-Salicrup et al. 2001; Schnitzer 2005; Schnitzer et al. 2005), but the competition for light and its subsequent impact on the leaf-trait configuration of host trees is still poorly understood. Therefore, we conducted this study in a tropical dry scrub forest to examine the leaf functional traits of co-occurring evergreen and deciduous tree species, as well as how these species’ leaf functional traits respond to liana infestation. In most aseasonal forests, where the competition for light is intense due to shading from neighboring trees, it will be difficult to neatly distinguish the impact of shade caused by the neighboring trees and liana loads. However, the sparsely distributed trees in the present study site are expected to face severe competition for light, primarily due to liana infestation. Therefore, liana colonization on host trees is considered the sole effector of variation in the light environment in this study. Moreover, the current study site provides a unique ecosystem with a mix of evergreen and deciduous species due to higher rainfall coupled with an extended dry season length, enabling us to understand the underlying mechanisms of different leaf habits coexisting in a given environment.

Although liana infestation on host trees is widely perceived to be detrimental to the host, we argue that the degree of impact varies among host trees with different light assimilation strategies (evergreen and deciduous). Furthermore, we hypothesize that liana colonization will affect the leaf functional traits of co-occurring deciduous and evergreen host trees differently. Specifically, we predict that evergreen trees will exhibit optimal plasticity in key leaf functional traits as a response to shade caused by liana colonization. On the other hand, we predict the acquisitive deciduous trees to exhibit more variations in leaf structural traits such as petiole elongation and leaf area expansion, which are often a maladaptive response to liana colonization. Therefore, the liana colonization will be more detrimental to acquisitive deciduous trees than to conservative evergreen host trees, as explained by their plasticity in leaf functional traits. Since lianas are seemingly increasing in abundance in tropical forests (Schnitzer and Bongers 2011; Vivek and Parthasarathy 2015), it is critical to understand their role in modifying the light environment and how this affects the host trees’ C-fixing abilities. The findings of such research models might contribute to a better understanding of the absolute impact of liana colonization on host trees’ productivity and aboveground competition.

## Materials and methods

### Study area

**Fig. 1:**
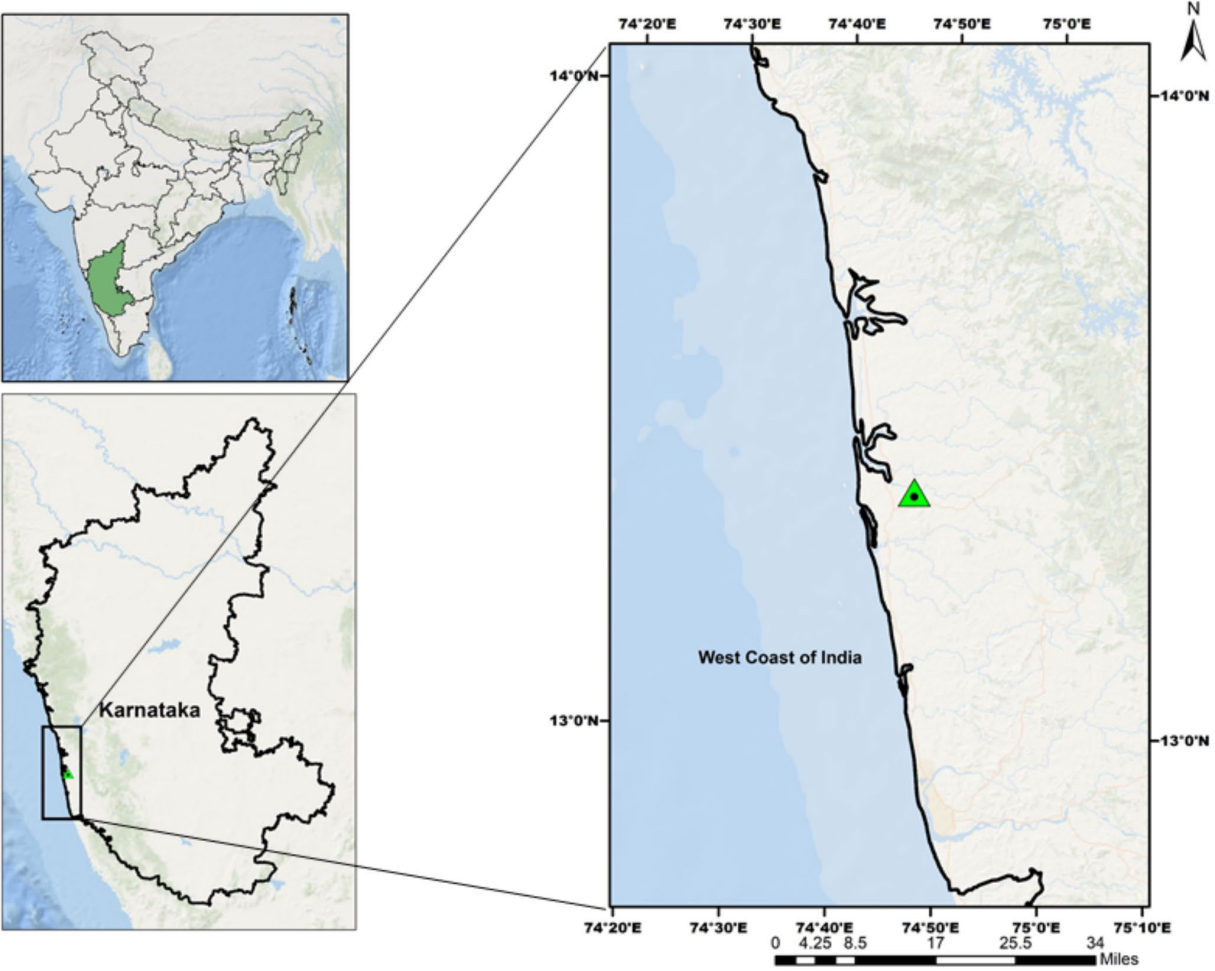
Map showing geographical location of the study area on the west coast of peninsular India

Leaf functional trait analysis was carried out in situ in a lateritic scrub forest on the West Coast of India (13° 22’ 19” N and 74° 47’ 00” E) (Fig. 1), dominated by deciduous and evergreen trees. Much of these forests have been cleared for cultivation and *Acacia* plantation, leaving only the remnants (Bhat 2014). The canopy is short (10–12 m) and composed of two to three-layered strata with a sparse distribution. The tree layer comprises *Terminalia paniculata*, *Carea arborea*, *Olea dioica, Macaranca peltata*, and *Holigarna arnottiana.* The study area also harbors *Acacia auriculiformis*, either deliberately planted or escaped from the adjoining plantations. *Getonia floribunda* and *Thunbergia grandiflora* are the predominant liana species, colonizing most of the trees at the edges, forming a dense carpet of leaves over the host trees’ crown. Species, including *Memecylon edule*, *Carissa spinarum*, and *Santalum album* dominated the lower canopy. The mean annual rainfall is 2693 mm, with more than 80% of the rainfall received during the South-West monsoon (June – September) with fewer sporadic showers of rain during the late summer (May). The study site also experiences an extended dry season length (5 months). The mean annual maximum and minimum temperatures correspond to 33 ℃ and 21 ℃ (Fick and Hijmans 2017, https://worldclim.org/).

### Species selection and sample collection

Based on the qualitative woody-species inventory, we selected a total of eight frequently occurring canopy trees, four each with contrasting leaf habits (Table S1). The individuals of the identified tree species were thoroughly screened for the presence/absence of lianas on the host trees’ crown. Lianas colonizing host trees’ crowns through lateral infestations (secondary host) were also considered along with the primary colonizers through the trunk. *Getonia floribunda*, a deciduous liana species, was a predominant colonizer that infested most of the trees in the study site, followed by *Thunbergia grandiflora*, an evergreen twiner, which colonized most of the trees through the edges of the patch. For each tree species, we identified five adult trees (GBH ≥ 60 cm) of similar size classes (60–90 cm GBH) with their crown homogenously shaded by the lianas (L^+^ category) and five individuals devoid of lianas (crown exposed to sunlight) on both trunk and crown (L^–^ category). The field collections were made during December-January, 2020-21, i.e., soon after the rainy season when deciduous lianas and trees maintained their full crown. A total of 11 leaf functional traits (Table S2) were estimated following Perez-Harguindeguy et al. (2016) and Vivek and Parthasarathy (2018). For each species under both categories, a total of 30 matured (fully expanded) and healthy leaves (free from folivore damage) were collected using a detachable tree pruner (up to 12 m). For L^+^ individuals, leaves were collected from the immediate branches beneath the liana layer (fully shaded). In the case of L^–^ individuals, fully-grown healthy sun leaves (fully- exposed) were collected for further analysis. The collected leaf samples were brought back to the laboratory in an insulated icebox within 30 min after the sample collection. The leaf samples were sequentially numbered with unique codes for different species using permanent markers at the abaxial side of the leaf. All the selected species had simple leaf types except *Millettia pinnata*, which had compound leaves. For *M. pinnata*, we collected the largest leaflet for leaf trait analysis following Perez-Harguindeguy et al. (2016). For each leaf sample, the petiole was removed, and petiole length (PL, cm) was measured before further processing. The leaf’s fresh weight (g) was measured using a top-pan balance (Shimadzu AUX-220, Japan). Leaf thickness (LT, mm) was measured using a digital micrometer at the intermediate point between the leaf lamina and midrib border, avoiding the secondary veins. The leaf area (one-sided area of a fresh leaf: LA, cm^2^) was measured using ImageJ (IJ) software (https://imagej.net/software/fiji/). The leaf samples were kept in a hot-air oven at 80 ℃ for 48 h. After measuring the dry weight (g), leaf samples were ground into a fine powder and stored in air-tight containers for further analysis. Mass-based leaf nitrogen concentration (N_mass_, mg g^− 1^) was analyzed using a CNHS analyzer (Elementar Analsensysteme GmbH, Germany) with triplicates. Specific leaf area (SLA, cm^2^ g^− 1^) was calculated as fresh leaf area (cm) / dry weight (g). Leaf tissue density (LTD, g cm^− 3^) was calculated as leaf mass per area (g cm^− 2^) / LT (cm). Leaf dry matter content (LDMC, mg g^− 1^) was calculated as leaf dry weight (mg) / leaf fresh weight (g). For chlorophyll estimation, 5–10 healthy and fully-grown leaves of similar age (for L^+^ and L^–^ categories) were collected from a minimum of two individuals per species to form a heterogeneous sample. For each species under the L^+^ and L^–^ categories, 1 g of the fresh leaf was randomly taken from the subsample with replicates. Leaf chlorophyll (CHL_a_ – Chlorophyll a and CHL_b_ – Chlorophyll b) was estimated following Arnon (1949) and Brown and Hooker (1977) using UV/Visible Spectrophotometer (Shimadzu UV-1800) at 645 and 663 nm respectively for CHL_b_ and CHL_a_. The following equations were used for the estimation of CHL_a_, CHL_b,_ and total chlorophyll (CHL_t_) expressed in mg g^− 1^ in the studied leaf samples:

CHL_t_ (mg g^− 1^) = 20.2 (A_645_) + 8.02 (A_663_) × 0.1     (1)

CHL_a_ (mg g^− 1^) = 12.7 (A_663_) – 2.69 (A_645_) × 0.1    (2)

CHL_b_ (mg g^− 1^) = 22.9 (A_645_) – 4.68 (A_663_) × 0.1    (3)

Where A_663_ and A_645_ are the absorbances at 663 and 645 nm, respectively and 0.1 is the conversion factor.

Nested ANOVA was performed to analyze if functional traits (response variable) of L^–^ deciduous and evergreen species (nested variable) vary between leaf habits (independent variable). This allowed for a comprehensive examination of the complex relationships between the factors and the response variables, taking into account the nesting structure of the data. Two-way analysis of variance (ANOVA) was used to test the variations in plant functional traits according to their habit (two levels: deciduous and evergreen) and exposure (two levels: L^+^ and L^–^) with interaction (habitat: exposure). Further, a post–hoc (Tukey HSD) test was performed for pairwise comparisons to identify the significant differences where ANOVA showed such variation. Parametric assumptions (normality and homogeneity of variance) were confirmed using the Shapiro–Wilk and Levene’s tests. Standardized Major Axis (SMA) regression was performed to understand the leaf trait-pair relationship between various leaf functional traits. All the statistical analyses were carried out in R 4.1.0 (R Development Core Team 2021) using the packages STAT (Bolar et al. 2019) and lmodel2 (Legendre and Oksanen 2018).

## Results

Results revealed that evergreen and deciduous leaf habits exhibited contrasting leaf functional strategies. Among evergreen species, *H. arnottiana* and *A. cardiosperma* registered the highest mean SLA in L^+^ and L^–^ categories, respectively (Table 1). While *A. cardiosperma* had the highest N_mass_ among the L^+^ evergreen species, *H. arnottiana* dominated in the L^−^ category. *H. arnottiana* also had the highest mean LA among evergreen species (104.77 ± 4.75 in L^−^ and 117.08 ± 5.23 cm^2^ in L^+^). Among deciduous species, *Z. mauritiana* had the highest mean SLA and N_mass_ and the lowest mean LA in both categories (Table 2). *C. arborea* registered the maximum LA among the deciduous species studied in L^+^ and L^–^ categories. *M. pinnata* accounted for the highest CHL_t_ among the four deciduous species in both L^+^ (2.305 mg g^− 1^) and L^–^ (1.182 mg g^− 1^) categories.

**Table 1:** Summary of the leaf functional trait analysis of four evergreen species in L^+^ and L^–^ categories. Values presented are mean and the standard error (SE) for 30 leaf samples/species/category. Chlorophyll and N_mass_ values are means of triplicates

Leaf trait	Species								Mean	
	*Holigarna arnottiana* Hook. f. (Anacardiaceae)		*Aporosa cardiosperma* (Gaertn.) Merr. (Phyllanthaceae)		*Carallia brachiata* (Lour.) Merr. (Rhizophoraceae)		*Olea dioica* Roxb. (Oleaceae)			
	L^−^	L^+^	L^−^	L^+^	L^−^	L^+^	L^−^	L^+^	L^−^	L^+^
LA (cm^2^)	104.77	117.08	49.85	50.08	33.66	48.12	56.64	47.4	61.23	65.67
	(4.75)	(5.23)	(2.75)	(1.92)	(0.93)	(1.25)	(1.69)	(2.14)	(13.24)	(14.85)
LT (mm)	0.332	0.194	0.254	0.202	0.347	0.334	0.331	0.152	0.32	0.22
	(0.01)	(0.00)	(0.00)	(0.00)	(0.00)	(0.04)	(0.00)	(0.00)	(0.02)	(0.03)
PL (cm)	0.4	0.53	0.56	0.57	0.46	0.57	0.22	0.23	0.41	0.48
	(0.02)	(0.02)	(0.01)	(0.02)	(0.01)	(0.01)	(0.00)	(0.00)	(0.06)	(0.07)
SLA (cm^2^ g^− 1^)	92.1	186.7	109.3	180.4	73.3	106.9	75.9	122.3	87.65	149.08
	(5.28)	(5.76)	(1.76)	(3.00)	(1.05)	(2.39)	(2.15)	(2.48)	(7.21)	(17.49)
LTD (g cm^3^)	0.327	0.277	0.36	0.275	0.393	0.28	0.398	0.539	0.37	0.34
	(0.02)	(0.00)	(0.01)	(0.00)	(0.01)	(0.00)	(0.01)	(0.01)	(0.01)	(0.06)
LDMC (mg g^− 1^)	469.79	369.92	452.41	328.2	390.56	365.79	504.78	465.92	454.39	382.46
	(7.39)	(8.04)	(4.02)	(3.75)	(2.55)	(4.64)	(8.61)	(7.90)	(20.70)	(25.43)
CHL_a_ (mg g^− 1^)	1.507	1.339	0.936	1.513	0.812	1.418	1.288	1.544	1.14	1.45
									(0.14)	(0.04)
CHL_b_ (mg g^− 1^)	0.536	0.648	0.586	1.25	0.268	0.723	0.566	1.039	0.49	0.92
									(0.06)	(0.12)
CHL_t_ (mg g^− 1^)	2.042	1.987	1.522	2.762	1.08	2.201	1.854	2.582	1.62	2.38
									(0.18)	(0.15)
CHL_a/b_ ratio	2.813	2.067	1.597	1.21	3.025	1.811	2.276	1.485	2.43	1.64
									(0.28)	(0.16)
N_mass_ (mg g^− 1^)	20.004	23.93	19.732	25.43	14.614	20.05	14.032	19.89	17.1	22.33
									(1.39)	(1.21)

L^+^ – tree crown colonized by lianas; L^–^ – tree crown free from lianas; LA – one-sided leaf area; LT – leaf thickness; PL – petiole length; SLA – specific leaf area; LTD – leaf tissue density; LDMC – leaf dry matter content; CHL_a_ – chlorophyll a; CHL_b_ – chlorophyll b; CHL_t_ – total chlorophyll; CHL_a/b_ – chlorophyll a/b ratio; N_mass_ – mass-based leaf nitrogen concentration

**Table 2:** Summary of the leaf functional trait analysis of four deciduous species in L^+^ and L^–^ categories. Values presented are mean and the standard error for 30 leaf samples/species/category. Chlorophyll and N_mass_ values are means of triplicates

Leaf trait	Species								Mean	
	*Careya arborea* Roxb. (Lecythidaceae)		*Terminalia paniculata* Roth (Combretaceae)		*Millettia pinnata* (L.) Panigr. (Fabaceae)		*Ziziphus mauritiana* (Rhamnaceae)			
	L^−^	L^+^	L^−^	L^+^	L^−^	L^+^	L^−^	L^+^	L^−^	L^+^
LA (cm^2^)	248.21	280.24	69.8	99.3	60.646	84.58	10.61	14.69	97.32	119.7
	(7.19)	(12.40)	(6.07)	(2.83)	(3.56)	(3.97)	(0.33)	(0.69)	(44.99)	(49.02)
LT (mm)	0.31	0.3	0.26	0.23	0.19	0.15	0.16	0.12	0.23	0.2
	(0.00)	(0.00)	(0.00)	(0.01)	(0.00)	(0.00)	(0.00)	(0.00)	(0.03)	(0.03)
PL (cm)	0.97	1.696	0.397	0.701	2.35	3.894	0.26	0.623	0.99	1.73
	(0.02)	(0.02)	(0.01)	(0.01)	(0.05)	(0.10)	(0.05)	(0.02)	(0.41)	(0.66)
SLA (cm^2^ g^− 1^)	120.26	148.27	113.12	108.67	147.7	215.53	174.01	226	138.77	174.62
	(7.13)	(3.63)	(3.56)	(2.31)	(6.28)	(5.14)	(6.28)	(2.30)	(12.05)	(24.18)
LTD (g cm^3^)	0.27	0.226	0.35	0.396	0.36	0.316	0.37	0.363	0.33	0.33
	(0.00)	(0.01)	(0.02)	(0.01)	(0.01)	(0.01)	(0.01)	(0.00)	(0.02)	(0.03)
LDMC (mg g^− 1^)	281.16	294.07	318.25	418.11	377.74	410.29	279.82	269.71	314.24	348.05
	(7.66)	(9.39)	(20.36)	(7.73)	(5.83)	(3.06)	(5.83)	(3.28)	(19.89)	(33.39)
CHL_a_ (mg g^− 1^)	0.702	0.916	0.89	1.467	0.879	1.791	0.88	1.194	0.84	1.342
									(0.04)	(0.16)
CHL_b_ (mg g^− 1^)	0.197	0.316	0.143	0.413	0.303	0.514	0.179	0.43	0.2	0.418
									(0.03)	(0.04)
CHL_t_ (mg g^− 1^)	0.899	1.231	1.033	1.879	1.182	2.305	1.066	1.623	1.045	1.76
									(0.05)	(0.20)
CHL_a/b_ ratio	3.566	2.902	3.555	6.211	2.907	3.484	4.907	2.777	3.73	3.84
									(0.36)	(0.70)
N_mass_ (mg g^− 1^)	22.982	23.41	27.652	28.55	26.96	29.35	37.24	39.77	28.71	30.27
									(2.62)	(2.97)

Abbreviations for leaf traits as in Table 1

Within the L^–^ category, deciduous species had significantly higher mean SLA (*P* < 0.05), N_mass_ (*P* < 0.05), and Chl_a/b_ ratio (*P* < 0.05), whereas the evergreen species had significantly higher mean LTD (*P* < 0.05) CHL_a_, CHL_b_, and CHL_t_ (*P* < 0.05) than the deciduous species (Table 3). Other parameters LA, LT, PL, and LDMC did not vary significantly between evergreen and deciduous species (*P* > 0.05).

**Table 3:** Results of the nested ANOVA to check for the significant difference in leaf functional traits between evergreen (L^–^) and deciduous (L^–^) species

Leaf trait	Leaf habit	Mean	SD	SE	*F*	*P*
LA (cm^2^)	Evergreen	61.2	26.4	13.2	0.025	0.879
	Deciduous	97.3	89.9	44.9		
LT (mm)	Evergreen	0.32	0.04	0.02	0.53	0.494
	Deciduous	0.23	0.06	0.03		
PL (cm)	Evergreen	0.41	0.12	0.06	3.18	0.125
	Deciduous	0.99	0.93	0.41		
SLA (cm^2^ g^− 1^)	Evergreen	87.6	14.4	7.21	8.20	0.028
	Deciduous	138.7	24.1	12.1		
LTD (g cm^3^)	Evergreen	0.37	0.03	0.01	8.198	0.021
	Deciduous	0.33	0.04	0.02		
LDMC (mg g^− 1^)	Evergreen	454.3	41.3	20.7	0.64	0.454
	Deciduous	314.2	39.7	19.8		
CHL_a_ (mg g^− 1^)	Evergreen	1.14	0.28	0.14	12.18	0.002
	Deciduous	0.98	0.29	0.14		
CHL_b_ (mg g^− 1^)	Evergreen	0.49	0.13	0.06	45.67	0.000
	Deciduous	0.27	0.09	0.05		
CHL_t_ (mg g^− 1^)	Evergreen	1.62	0.37	0.18	27.75	0.000
	Deciduous	1.26	0.37	0.19		
CHL_a/b_ ratio	Evergreen	2.43	0.55	0.28	25.36	0.000
	Deciduous	3.73	0.73	0.36		
N_mass_ (mg g^− 1^)	Evergreen	17.1	2.78	1.39	51.07	0.000
	Deciduous	28.7	5.24	2.62		

SD – standard deviation; SE – standard error (*n* = 4); abbreviations for leaf traits as in Table 1

The two-way ANOVA test revealed that the liana colonization status had a significant impact on all the leaf functional traits studied, except for LA (Table 4). Similarly, the leaf habit explained significant variations among all traits except LA and LT. The comparison between L^+^ and L^–^ trees indicated that both evergreen and deciduous species registered higher SLA and higher Chlorophyll (CHL_a_, CHL_b_, and CHL_t_) in L^+^ over the L^–^ individuals (Tables 1 and 2). Among deciduous species, L^+^ trees registered higher LA, PL, and LDMC than the L^–^ individuals (Table 2), but those were similar between L^+^ and L^–^ evergreen trees as revealed by Tukey’s pairwise post-hoc test (Table 5). Moreover, the L^+^ evergreen trees had lower LT, LTD, and higher N_mass_ than the L^–^ individuals (Table 1), but those were similar between L^+^ and L^–^ deciduous trees as revealed by Tukey’s pairwise post-hoc test (Table 5).

**Table 4:** Summary of two–way ANOVA for effect of liana colonization status, leaf habit and liana colonization status × leaf habit on the studied leaf traits

		LA (cm^2^)	LT (mm)	PL (cm)	SLA (cm^2^ g^–1^)	LTD (g cm^3^)	LDMC (mg g^–1^)	CHL_a_ (mg g^–1^)	CHL_b_ (mg g^–1^)	CHL_t_ (mg g^–1^)	CHL_a/b_	N_mass_ (mg g^–1^)
Liana colonisation status	*F* *p*	2.08 0.15	32.07 0.00	16.36 0.00	117.34 0.00	37.99 0.00	19.42 0.00	41.79 0.00	74.20 0.00	68.24 0.00	22.75 0.00	11.05 0.001
Leaf habit	*F* *p*	3.63	0.01	273.56	29.23	28.42	158.05	12.14	103.99	53.177	81.79	68.52
		0.06	0.93	0.00	0.00	0.00	0.00	0.001	0.00	0.00	0.00	0.00
Liana colonisation status: Leaf habit	*F* *p*	0.19	21.28	2.25	51.92	11.28	15.6	2.36	0.37	0.38	0.29	5.27
		0.66	0.00	0.13	0.00	0.00	0.00	0.00	0.54	0.00	0.59	0.03

Abbreviations for leaf traits as in Table 1

**Table 5:** Summary of pairwise comparisons generated with post-hoc test (Tukey test) based on two–way ANOVA

	L^+^ Decd vs. L^–^ Decd	L^–^ Eveg vs. L^–^ Decd	L^+^ Evrg vs. L^–^ Decd	L^–^ Evrgr vs. L^+^ Decd	L^+^ Evrg vs. L^+^ Decd	L^+^ Evrg vs. L^–^ Evrg
LA (cm^2^)	0.000	0.000	0.000	0.000	0.000	0.988
LT (mm)	0.879	0.007	0.000	0.000	0.005	0.000
PL (cm)	0.000	0.000	0.000	0.000	0.000	0.274
SLA (cm^2^ g^–1^)	0.040	0.000	0.000	0.000	0.580	0.000
LTD (g cm^3^)	0.195	0.000	0.935	0.000	0.503	0.000
LDMC (mg g^–1^)	0.000	0.000	0.000	0.000	0.000	0.988
CHLa (mg g^–1^)	0.000	0.004	0.000	0.166	0.520	0.005
CHLb (mg g^–1^)	0.000	0.000	0.000	0.679	0.000	0.000
CHLt (mg g^–1^)	0.000	0.000	0.000	0.902	0.000	0.000
CHLa/b	0.022	0.000	0.000	0.020	0.000	0.002
N_mass_ (mg g^–1^)	0.885	0.000	0.005	0.000	0.000	0.001

Abbreviations for leaf traits as in Table 1

**Fig. 2:**
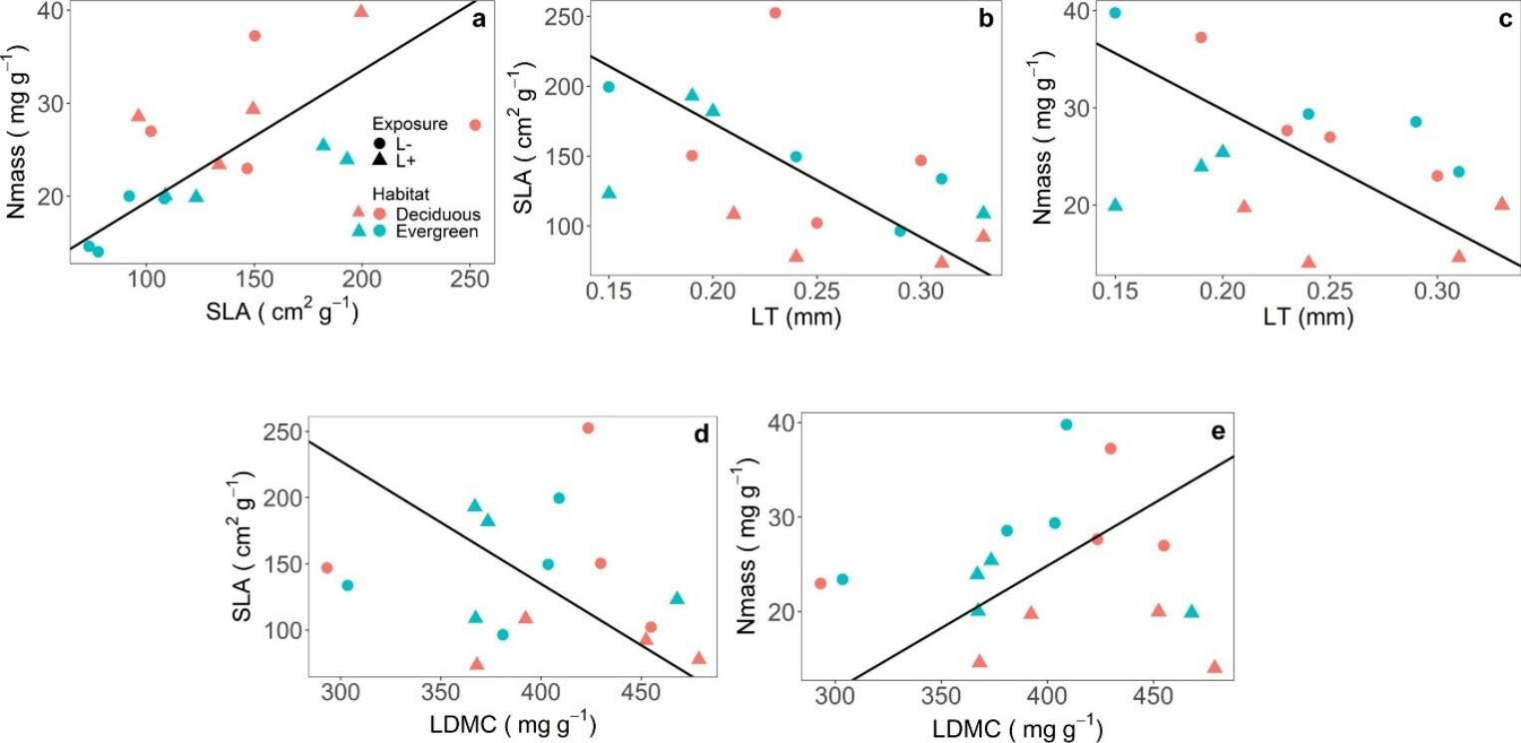
Standardized major axis (SMA) regression for trait-pair relationship between different trait-pairs using pooled data of L^+^ and L^–^ categories (evergreen and deciduous species). **a** SLA vs. N_mass_, **b** LT vs. SLA, **c** LT vs. N_mass_, **d** LDMC vs. SLA and **e** LDMC vs. N_mass_. Data points are species means with *n* = 16 per species. Summary of SMA is given in Table S3. Abbreviations for leaf traits as in Table 1

The leaf trait-pair relationship analysis using the pooled data of L^+^ and L^–^ categories revealed that there was a positive correlation between SLA and N_mass_ (*r*^*2*^ = 0.66; *P* < 0.001) (Table S3, Fig. 2a). LT was negatively correlated with SLA (*r*^*2*^ = 0.66; *P* < 0.001) (Fig. 2b) and N_mass_ (*r*^*2*^ = 0.55; *P* < 0.01) (Fig. 2c). Similarly, LDMC was negatively correlated with SLA (*r*^*2*^ = 0.30; *P* < 0.05) (Fig. 2d) and positively correlated with N_mass_ (*r*^*2*^ = 0.42; *P* < 0.001) (Fig. 2e). There was no correlation observed between N_mass_ and CHL_a, b, t_. Similarly, PL with LA and LT did not show any correlation. SMA results explained a similar trend when data were analyzed for only L^–^ categories (Table S3).

## Discussion

### Co-occurrence of evergreen and deciduous species

Interspecific variation in plant functional traits forms the basis of species co-existence in natural ecosystems. The evergreen and deciduous species (L^–^) in the present study showed contrasting leaf functional strategies for C-assimilation. The deciduous species with higher SLA and N_mass_ coupled with relatively thinner leaves and lower LDMC displayed a set of traits confirming their acquisitive strategy with a shorter leaf lifespan. In contrast, the evergreen species exhibited a conservative plan with relatively thicker leaves, and lower SLA and N_mass_ in the present study. Our results are consistent with the findings of recent studies, for example, Ellsworth and Sternberg (2016), Jiang et al. (2016), and de Souza et al. (2020). Deciduous species tend to invest more in active photosynthetic machinery under high light availability to compensate for the short growing season and sustain higher photosynthetic rates (Niinemets et al. 2004; Wright et al. 2005). Therefore, deciduous species generally exhibit relatively higher potential growth rates and higher photosynthetic rates than evergreen species (Cornelissen et al. 1996; Reich et al. 1992; Worbes et al. 2013). Evergreen species can compensate for their lower photosynthetic rates by assimilating carbon throughout the year (Aerts 1995; Baldocchi et al. 2010; Givnish 2002). Therefore, there is an inevitable trade-off between maximizing carbon fixation and leaf longevity in evergreen and deciduous species. The shorter leaf life span in deciduous species can be attributed to the strategy to avoid transpiration during the period of water storage and favour high tissue leaf water potential (Borchert et al. 2002; Levitt 1980; Markesteijn and Poorter 2009), at least in the seasonally dry tropical forests. In contrast, evergreen species retain their leaves during the entire year across seasons, confirming their ability to compensate for transpiration loss through efficient root hydraulics (Ackerly 2004; Borchert 1994).

Although evergreen species had higher LT and LDMC than deciduous species, the difference between the two distinct leaf habits studied was not significant, contradicting the findings of Castro-Díez et al. (2000) and Kröber et al. (2015). This may be explained by the smaller sample size of species and the greater variation in LT among the deciduous species. *C. arborea*, for example, had nearly twice the leaf thickness of *Z. mauritiana*, indicating the effect of species-specific values rather than group-specific values. However, our results correspond with Burrows (2001), and de Souza (2020), who reported a similar trend in the seasonally dry tropical forests. Similarly, PL and LA did not show significant variation between the two leaf habits. However, deciduous species had relatively higher PL and LA than the evergreen species in the studied site. The higher PL and LA can be linked to the strategy for enhanced resource acquisition by maximizing the net photosynthetic area available for light capture and avoiding shade (Falster and Westoby 2003; Weijschedé et al. 2006; Yamada et al. 2000). Overall, there was a significant difference between evergreen (L^–^) and deciduous (L^–^) species in seven of the 11 traits examined. Although functional traits are known to vary with leaf habit (e.g., Krober et al. 2015), the convergence of other studied traits suggests the underlying mechanisms of species co-existence and resource partitioning in a landscape dominated equally by evergreen and deciduous species.

### Acclimation to liana-mediated shade in evergreen and deciduous species

The SLA and chlorophyll components of evergreen and deciduous species responded similarly to liana colonization, indicating their importance as potential predictors of changing light environments irrespective of the species’ leaf habit. While the SLA is highly correlated with mass-based photosynthesis and respiration rates (Wright et al. 2004), leaf chlorophyll is also one of the key parameters directly related to photosynthetic potential and may provide valuable information on the physiological status (Croft et al. 2017; Riccardi et al. 2014). The relatively higher chlorophyll content (a, b, and total chlorophyll) in the L^+^ leaves of evergreen and deciduous species is possibly due to a greater level of photooxidation under very high light intensities than in the shaded environments (Kramer and Kozlowski 1979). Ntawuhiganayo et al. (2020) also reported the higher chlorophyll content in shade-tolerant species and their ability to increase it further upon shading. However, the higher chlorophyll content in shade-tolerant species may not reflect positively on the quantum yield efficiency (Dusenge et al. 2015). Therefore, the higher chlorophyll content in the L^+^ leaves does not necessarily involve enhanced leaf carbon gain. The significantly higher Chlorophyll b in the L^+^ leaves can be because of the strategy to absorb light in the blue spectrum, which is prevalent in shaded environments. Therefore, leaves acclimated to low-light environments tend to have a lesser chlorophyll a/b ratio (Lichtenthaler et al. 2007). The size of the photosystem II (PSII) antenna in shaded environments could also be attributed to the lower level of the chlorophyll a/b ratio in the L^+^ leaves, specifically of evergreen species (Anderson et al. 1995; Tanaka and Tanaka 2000). Sun leaves have higher chlorophyll a/b ratio than shade leaves, indicating a lower number of light-harvesting chlorophyll a + b-binding antenna complexes (Anderson et al. 1995). However, a few studies have reported the opposite trend (e.g., Falbel et al. 1996; Zivcak et al. 2014), indicating its impact at the species level rather than at the community level.

Within evergreen species, L^+^ and L^–^ categories showed a significant difference in SLA and N_mass_. The higher SLA and N_mass_ in the L^+^ category reflect the shade-tolerant characteristics of the evergreen species that help in maximizing the carbon gain with minimum expenditure. Therefore, the shaded leaves of evergreen species are more efficient under low light conditions with higher SLA coupled with the specialized anatomy of single-layered palisade parenchyma that results in the formation of thin leaves (Givnish 1988; Lombardini et al. 2009). The present study also reported relatively thinner leaves in the L^+^ category, which further signifies the importance of conserving carbon in low-light environments. However, the L^–^ leaves with comparatively thicker leaves are associated with well-developed mesophyll tissues, possibly to avoid photo-inhibitory damage as they get exposed to higher light intensities (Taiz and Zeiger 2006). Furthermore, studies with woody species showed thicker mesophyll tissues with reduced intercellular spaces in plants grown in bright sunlight, compared to plants grown in shade (Nakazono et al. 2001; Piel et al. 2002). The increased LT at higher irradiance can also enhance the photosynthetic efficiency (Atanasova et al. 2003). PL and LA, both associated with the enhanced light acquisition strategies, did not show any significant variation between L^+^ and L^–^ categories of evergreen species, which strongly reflects their conservative response in petiole elongation and leaf area expansion.

In contrast to the evergreen species, the deciduous species showed more plasticity in the structural traits (PL and LA), but not in LTD and N_mass_. For instance, the PL in the deciduous L^+^ category was close to two-fold higher than L^–^ leaves. Petiole length plays a dual role by enhancing the leaf light-harvesting efficiencies and influencing the leaf angle (Weijschedé et al. 2006). The PL elongation in the L^+^ category should have been beneficial if it leads to enhanced resource acquisition, as petiole length elongation can improve resource acquisition by avoiding shade (Weijschedé et al. 2006). However, in the present study, the petioles of the L^+^ category did not reach the better-lit areas, leading to decreased light-harvesting efficiencies despite investing more in the non-photosynthetic throwaway biomass. Therefore, the plastic responses in the petiole length to liana proliferation can be increasingly expensive in biomass investments. In contrast to our observations, Niinemets et al. (2004) showed that PL was higher in the exposed areas than in the shaded regions of dense poplar plantations. Therefore, the other unstudied whole-plant architectural parameters might also play a role in the petiole elongation. L^+^ deciduous species also had a relatively higher leaf area than the L^–^ categories. These plastic responses in PL and LA confirm the shade-intolerant characteristics of the deciduous species studied. Although the ontogenic changes in PL and LA are known to reduce the negative fitness against shading (Takenaka 1994; Yamada et al. 2000), they were associated only with resource expenditure as throwaway biomass in the present study.

## Conclusion

The findings of the present study supported our hypothesis that liana colonization will have a differential impact on the functional traits of host trees with contrasting leaf habits. Overall, the evergreen species showed plastic variations in the functional traits between L^+^ and L^–^ categories under varying light environments. Such variation in the leaf traits can enhance leaf photosynthesis at a specific irradiance level (Givnish 1988). The deciduous species, however, exhibited more plastic response towards structural traits, which could be a maladaptation. The allocation strategy of evergreen species can be considered successful in the present study concerning carbon gain under low-light conditions. For instance, Ntawuhiganayo et al. (2020) found only a minimal decline in the growth of the shade-tolerant species under low-light conditions than the shade-intolerant species. Thus, evergreen species showed better acclimation to the liana colonization than the deciduous species in terms of plastic responses in major traits and better energy allocation between photosynthetic and non-photosynthetic tissue to enhance carbon gain without compromising the structural stability of their leaves. However, deciduous species may have a competitive advantage in L^–^ categories with the legacies of efficient photosynthetic machinery over the evergreen species, given that water is not a constraining factor in the study site. Liana infestation on host trees’ crowns may decrease the ratio of the sun: shade leaves and the net amount of leaf area exposed to diffuse and direct sunlight, thus reducing the carbon gain per unit leaf area. Although liana colonization, in general, is considered harmful for the host trees, it may be more detrimental for fast-growing/shade-intolerant species (Visser et al. 2018). On the other hand, lianas may be beneficial for some of the shade-tolerant species, allowing them to grow in the shade and protect them from photoinhibition because shade-tolerant species may also be sun-intolerant (Ntawuhiganayo et al. 2020). Nevertheless, this impact of lianas on the leaf functional traits of host trees can have a significant impact on the whole forest carbon assimilation rates, particularly in the tropics, where they are abundant. We recommend the need for long-term observational studies to understand the dynamics in canopy occupancy by lianas and its impact on the host trees’ productivity.

## Electronic supplementary material

Below is the link to the electronic supplementary material.

Supplementary Material 1

## Data Availability

All data generated or analyzed during this research work are included in this manuscript.
