# Supplementary material for: Differential impact of liana colonization on the leaf functional traits of co-occurring deciduous and evergreen trees in a tropical dry scrub forest
Source: J Plant Res. 2023 May 31;136(5):679–90. doi: 10.1007/s10265-023-01474-4 (PMC10421778; doi:10.1007/s10265-023-01474-4)
Supplement: Supplementary file 1 — Supplementary Material 1 [file 10265_2023_1474_MOESM1_ESM.pdf]

Journal: Journal of Plant Research

Title: Differential impact of liana colonization on the leaf functional traits of co-occurring deciduous and evergreen trees in a tropical dry scrub forest

Authors: Vivek Pandi[1*] . Kanda Naveen Babu[2,3] . Ashaq Ahmad Dar[2]

Affiliations:

[1]Manipal Centre for Natural Sciences, Centre of Excellence, Manipal Academy of Higher Education (MAHE), Madhava Nagar, Manipal, 576 104, Udupi, Karnataka, India.

[2]Department of Ecology and Environmental Sciences, School of Life Sciences, Pondicherry University, Puducherry 605 014, India

[3]Department of Ecology, French Institute of Pondicherry, Pondicherry 605 001, India

Corresponding author:

Vivek Pandi

Manipal Centre for Natural Sciences, Centre of Excellence, Manipal Academy of Higher Education (MAHE), Madhava Nagar, Manipal, 576 104, Udupi, Karnataka, India.

Email: vivek.pandi@manipal.edu

Table S1 List of tree species selected for the study based on their leaf type and liana colonization status.

Species	Family	Leaf form	Leaf type
Holigarna arnottiana Hook. f.	Anacardiaceae	Simple	Evergreen
Aporosa cardiosperma (Gaertn.) Merr.	Phyllanthaceae	Simple	Evergreen
Carallia brachiata (Lour.)	Rhizophoraceae	Simple	Evergreen
Olea dioica Roxb	Oleaceae	Simple	Evergreen
Careya arborea Roxb.	Lecythidaceae	Simple	Deciduous
Terminalia paniculata Roth	Combretaceae	Simple	Deciduous
Millettia pinnata (L.) Panigr.	Fabaceae	Compound	Deciduous
Ziziphus mauritiana Lam.	Rhamnaceae	Simple	Deciduous

Table S2 List of leaf functional traits investigated, including abbreviations, units of measurement, and ecological significance.

Leaf trait	Abbreviation	Unit	Ecological relevance/significance
Specific Leaf Area	SLA	$cm^2\ g^{-1}$	Scale positively with relative plant growth rate
Leaf Tissue Density	LTD	$g\ cm^{-3}$	Determines plant resource acquisition strategies. Negatively related to plant growth rate
Leaf Area	LA	cm^2	Reflects the ecological strategy of the plant in relation to its climate, geography, nutrient, disturbance and phylogenetic factors
Petiole length	PL	cm	Plays a critical role in the spatial positioning of the leaf in relation to the availability of light
Leaf Dry Matter Content	LDMC	$mg\ g^{-1}$	LDMC has been shown to correlate negatively with potential RGR and positively with leaf lifespan
Leaf Thickness	LT	mm	Reflects the number and thickness of mesophyll layers and is driven largely by climatic variables (LT tend to be higher in sunnier environments)
Mass-based Leaf Nitrogen Concentration	N_{mass}	$mg\ g^{-1}$	Positively related to light-saturated photosynthetic rate
Chlorophyll a	CHL_a	$mg\ g^{-1}$	Reflects the quality and quantity of the light environment. Positively related to sunnier environment
Chlorophyll b	CHL_b	$mg\ g^{-1}$	Reflects the quality and quantity of the light environment. Positively related to shaded environment
Total Chlorophyll	CHL_t	$mg\ g^{-1}$	Reflects leaf photosynthetic capacity. Positively related to the leaf photosynthetic capacity
Chlorophyll a/b ratio	$CHL_{a/b}$	NA	Indicates the extent of shade tolerance in plants. A higher a/b ratio reflects greater shade tolerance

Table S3 Summary of the standardized major axis (SMA) regression between different trait-pairs using pooled data of L^+ and L^- categories (evergreen and deciduous species) and only L^- category.

Trait-pair	*n*	r^2	*P*	Slope	Intercept
Pooled data (L^+ and L^-)					
SLA vs N_{mass}	16	0.665	0.0001	0.82970	-0.37994
LT vs SLA	16	0.666	0.0001	-1.00446	1.47081
LT vs N_{mass}	16	0.547	0.001	-0.83341	0.84040
LDMC vs N_{mass}	16	0.420	0.001	0.130	-27.790
LDMC vs SLA	16	0.302	0.027	-1.69828	6.47130
Only L^-					
SLA vs N_{mass}	8	0.897	0.0003	0.91576	0.81011
LT vs SLA	8	0.826	0.0017	-1.0328	1.43692
N_{mass} vs LT	8	0.727	0.007	-1.1272	0.68446
LDMC vs N_{mass}	8	0.549	0.035	1.4324	5.02740
LDMC vs SLA	8	0.459	0.064	-1.3118	5.41404

Abbreviations for leaf traits as in Online Resource 2
